# The *cis-trans* binding strength defined by motif frequencies facilitates statistical inference of transcriptional regulation

**DOI:** 10.1186/s12859-019-2732-6

**Published:** 2019-05-01

**Authors:** Yance Feng, Sheng Zhang, Liang Li, Lei M. Li

**Affiliations:** 1 National Center of Mathematics and Interdisciplinary Sciences, Academy of Mathematics and Systems Science, Chinese Academy of Sciences, Beijing, 100190 China; 2 University of Chinese Academy of Sciences, Beijing, 100049 China; 3 Center for Excellence in Animal Evolution and Genetics, Chinese Academy of Sciences, Kunming, 650223 China

**Keywords:** BASE, Statistical inference, Transcriptional regulation, PUFA, DHA, EPA, Binding strength

## Abstract

**Background:**

A key problem in systems biology is the determination of the regulatory mechanism corresponding to a phenotype. An empirical approach in this regard is to compare the expression profiles of cells under two conditions or tissues from two phenotypes and to unravel the underlying transcriptional regulation. We have proposed the method BASE to statistically infer the effective regulatory factors that are responsible for the gene expression differentiation with the help from the binding data between factors and genes. Usually the protein-DNA binding data are obtained by ChIP-seq experiments, which could be costly and are condition-specific.

**Results:**

Here we report a definition of binding strength based on a probability model. Using this condition-free definition, the BASE method needs only the frequencies of *cis*-motifs in regulatory regions, thereby the inferences can be carried out in silico. The directional regulation can be inferred by considering down- and up-regulation separately. We showed the effectiveness of the approach by one case study. In the study of the effects of polyunsaturated fatty acids (PUFA), namely, docosahexaenoic (DHA) and eicosapentaenoic (EPA) diets on mouse small intestine cells, the inferences of regulations are consistent with those reported in the literature, including PPARα and NFκB, respectively corresponding to enhanced adipogenesis and reduced inflammation. Moreover, we discovered enhanced RORA regulation of circadian rhythm, and reduced ETS1 regulation of angiogenesis.

**Conclusions:**

With the probabilistic definition of *cis-trans* binding affinity, the BASE method could obtain the significances of TF regulation changes corresponding to a gene expression differentiation profile between treatment and control samples. The landscape of the inferred *cis-trans* regulations is helpful for revealing the underlying molecular mechanisms. Particularly we reported a more comprehensive regulation induced by EPA&DHA diet.

**Electronic supplementary material:**

The online version of this article (10.1186/s12859-019-2732-6) contains supplementary material, which is available to authorized users.

## Background

The central dogma is the core model of molecular biology. According to this dogma, in a cell DNA is transcribed into RNA, and the coding RNA is translated into various kinds of proteins with specific biological functions such as signaling, transport, molecular binding, etc. In recent decades the framework of DNA → RNA → protein has been extended by the discoveries of many kinds of non-coding RNAs such as microRNA, long non-coding RNA, and by the discoveries of many kinds of molecular modifications such phosphorylation and methylation. All these molecules and modifications play important roles in the cellular networks.

When certain transcription factor(s) bind onto the specific short sequence motifs in the upstream promoter regions of a DNA segment, they can recruit polymerase and the transcription starts. The short sequence motifs and the factors are occasionally referred to as “*cis*” elements and “*trans*” factors. The coupling of “*cis*” elements and “*trans*” factors is specific, although not necessarily unique or exclusive. Mathematically, a “*cis*” motif could be represented by a joint multinomial distribution, each component of which corresponds to a DNA position in the short sequence. Such a probabilistic model can be transformed into a position weight matrices (PWM). The databases of “*cis*” motifs, their PWMs together with their corresponding “*trans*” factors include TRANFAC [1], JASPAR [2]. The binding strength between a transcription factor and a DNA segment could be evaluated in silico using its motif PWM and the likelihood method. In contrast, the binding between transcription factors and DNA sequences in vivo or in vitro can be measured by the technique of ChIP-chip or ChIP-seq [3]. The protein-DNA binding results from ChIP-chip or ChIP-seq are conditional on environments and on status of the cells in the experiments while the binding affinity estimated in silico is condition-free. In recent years, it is shown that chromatin accessibility data are very valuable for predicting the gene expression levels [4]. Nevertheless, the data of chromatin accessibility is condition-specific too.

A key problem in systems biology is the determination of the regulatory mechanism corresponding to a phenotype. A typical situation is to compare the expression profiles of cells under two conditions or tissues from two phenotypes and to figure out the underlying molecular mechanism empirically. Around the year of 2006, we postulated the problem of statistically inferring the effective regulatory factors that are responsible for the gene expression differentiation profiles based on the binding data between the factors and genes. To achieve the goal, C Cheng et al. [5] proposed a method that statistically infers activity changes of transcription factors, referred to as BASE (Binding association with sorted expression differentiation). The notion of BASE is different from those methods rooted in the Pearson correlation coefficient. Later C Cheng and LM Li [6] applied the same idea to infer the effective regulatory activities of miRNAs by integrating expression profile data with miRNA target predictions. In the version of BASE2.0 [7], we simplified the calculations of *p*-values and made the transcriptional inferences for the up- and down-regulated genes separately. The inference of BASE hinges on the binding data between factors and the regulatory regions of genes. It is ideal if ChIP-seq data of the corresponding conditions are available. In the absence of ChIP-seq data, we still hope to provide a sensible definition of binding strength using only the information of genome sequences and motif PWMs, which are condition-free. In our initial effort, we consider the output of the MAST software [8], which offers the *p*-value of a motif search within a sequence. We take the negative logarithm of the *p*-values as the definition of binding strengths. In this article, we propose a probabilistic model that defines the binding strength by a chance. According to this model, it is the motif frequency occurring in the regulatory regions that matters most in the transcriptional inference. We illustrate the effectiveness of the method by one case study.

## Methods

### A recapitulation of BASE 2.0

Since the proposal of BASE, we have modified its steps in several aspects. For the sake of clarity, a recap of the BASE 2.0 procedure is provided in the scenario of control and treatment. Suppose that we have the gene expression profiles of two samples, denoted by {*e*_*ij*_, *i* = 1, 2, …‚*N*, *j* = 0, 1}, namely, there are *N* genes, the value 0 and 1 of the label *j* respectively correspond to the control and treatment sample, and *e*_*ij*_ is the expression abundance of the *i*-th gene from the *j*-th sample, after appropriate preprocessing. As usual, we take the gene expression changes by the logarithm of the fold changes, i.e. *d*_*i*_ = *log*_*e*_*e*_*i*1_/*e*_*i*0_. Denote the expression differentiation vector of the *N* genes by (*d*_1_, *d*_2_,  … , *d*_*N*_). Hereafter, we consider the up- and down-regulated gene expression profiles separately. Let, ${d}^{+}=\left({d}_1^{+},{d}_2^{+},\dots, {d}_N^{+}\right),$ ${d}^{-}=\left({d}_1^{-},{d}_2^{-},\dots, {d}_N^{-}\right)$, where ${d}_i^{+}=\max \left\{{d}_i,0\right\}$, ${d}_i^{-}=\max \left\{-{d}_i,0\right\}$. Let the binding strength vector of a transcription factor *T* with the promoter regions of the *N* genes be *b* = (*b*_1_, *b*_2_,  … , *b*_*N*_). We first consider the inference of up-regulation using *d*^+^, and the inference of down-regulation using *d*^−^ can be made similarly. The method BASE 2.0 include the following steps,

1. Sort the elements of *d*^+^ in the descending order, and denote the result by${d}_{\pi (1)}^{+}\ge {d}_{\pi (2)}^{+}\ge \cdots \ge {d}_{\pi (N)}^{+}$, where *π* = (*π*(1), *π*(2), ⋯‚*π*(*N*)) is a permutation of (1, 2, ⋯, N) satisfying the above inequalities.
2. Compute two cumulative distribution functions as follows:
  $$ {f}_0(i)={\sum}_{j=1}^i{d}_{\pi (j)}/{\sum}_{j=1}^N{d}_{\pi (j)}, $$

and

$$ {f}_1(i)={\sum}_{j=1}^i{d}_{\pi (j)}^{+}{b}_{\pi (j)}/{\sum}_{j=1}^N{d}_{\pi (j)}^{+}{b}_{\pi (j)}, $$

for *i* = 1, 2, ⋯‚*N*.

[3] Calculate the BASE score for this motif:
  $$ \delta =\underset{1\le i\le N}{\max}\left[{f}_1(i)-{f}_0(i)\right] $$
[4] Calculate the *p*-value of the above the BASE score *δ* via permutation. That is, we randomly generate a permutation of (1, 2, ⋯, N), denoted by *λ* = (*λ*(1), *λ*(2), ⋯‚*λ*(*N*)); in Step [2], re-calculate *f*_1_(*i*) replacing *b*_*π*(*j*)_ by *b*_*λ*(*j*)_; in Step [3], re-calculate the BASE score *δ*^∗^; denote the scores resulted from *K* permutations by {*δ*^∗(1)^, *δ*^∗(2)^, ⋯ *δ*^∗(*K*)^}, then the *p*-value of the BASE score *δ* is evaluated as follows,
  $$ p=\frac{1}{K}\sum \limits_{k=1}^K\mathbf{1}\left({\delta}^{\ast (k)}>\delta \right), $$
  namely, the fraction of scores from permutations that are larger than the observed one.
[5] Rank the motifs/factors according to their significances, namely, *p-*values.

### A probability model for the definition of *cis-trans* binding strength

According to our observations, if a motif appears in the promoter region of a gene, it usually appears multiple times [9]. Thus, we consider a chance model for the binding event of a factor and a DNA segment. Given a regulatory factor *T*, the PWM of its *cis*-element motif, and upstream sequence *S* of a gene, we can run a kind of local alignment of the motif along *S*. A possible binding site is identified when the alignment score is above a certain threshold. Suppose *τ* binding sites are found in *S*. Furthermore, we assume the binding events of *T* with these *τ* sites are independent, and identical distributed Bernoulli trials with a binding probability *p*_0_. Then the probability that *T* binds to the region *S*, or more precisely, that *T* binds to at least one site is given by 1 − (1 − *p*_0_)^*τ*^. When *p*_0_ is sufficiently small, we can approximate it by

$$ 1-{\left(1-{p}_0\right)}^{\tau}\approx \tau {p}_0 $$

Consequently, we replace the binding vector by *b* ≈ *p*_0_(*τ*_1_, *τ*_2_, ⋯, *τ*_*N*_) Now the second equation in Step [2] becomes

$$ {f}_1(i)\approx {\sum}_{j=1}^i{d}_{\pi (j)}^{+}{\tau}_{\pi (j)}/{\sum}_{j=1}^N{d}_{\pi (j)}^{+}{\tau}_{\pi (j)} $$

Since *p*_0_ is a common term in both denominator and numerator, it disappeared in this equation. According to this probability model, the calculation of the BASE score only involves the motif frequencies in the regulatory regions. In other words, there is no need to estimate the binding probability *p*_0_ for each transcription factor if they are relatively small.

It is noted that the cumulative function *f*_1_(*i*) in Step [2] is scale-free with respect to both the expression differentiation vector *d* and the count vector *τ* = (*τ*_1_, *τ*_2_, ⋯, *τ*_*N*_). Similarly, the cumulative function *f*_1_(*i*) in (1) is as well scale-free with respect to the expression differentiation vector *d*. Thus, the BASE score shown in Step [3] is scale-free with respect to both the expression differentiation vector and the count vector. The scale-free property presents a kind of robustness in the BASE inference.

**Fig. 1:**
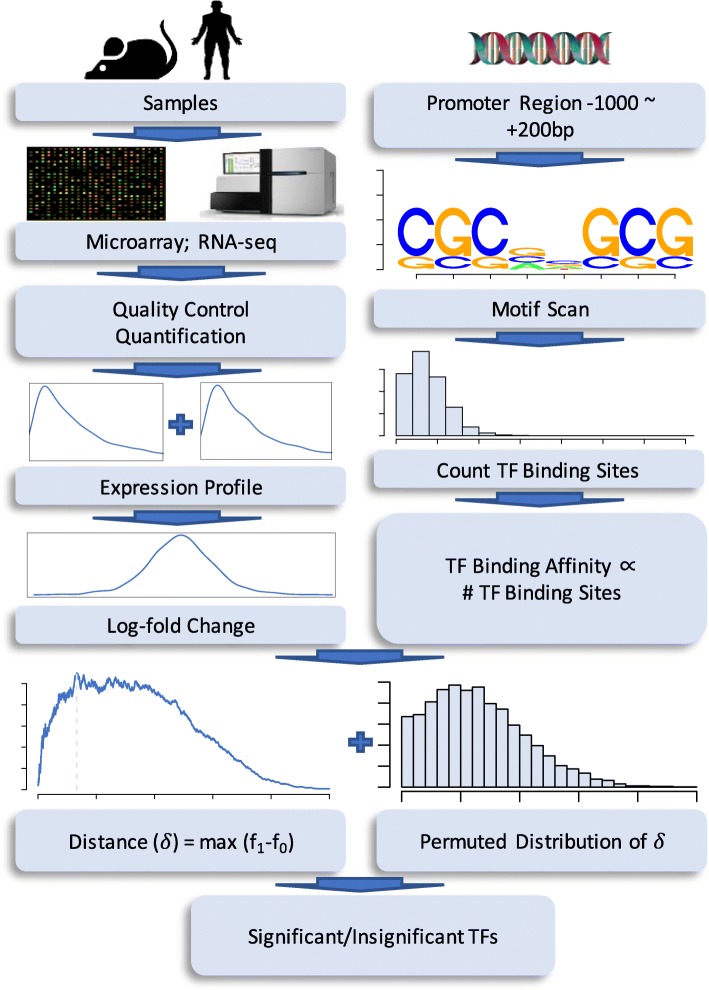
The workflow of the modified BASE using *cis-trans* binding strength defined by motif frequencies

Given the PWM of a *cis*-element, the BASE 2.0 procedure evaluates the statistical significance of its regulatory role by a *p*-value. If we would like to have strong control of the false discovery rate (FDR) over the multiple inferences, we could adopt the adjusted significances, or the q-values [10]. The current procedure of modified BASE is illustrated in Fig. 1.

### Counting TF binding sites

In light of the probabilistic definition of *cis-trans* binding strength, the calculation of BASE score, see the definition of *f*_1_(*i*), requires the counts of motif occurrences in the promoter regions of genes. Our in silico solution is to estimate the counts by the searching the motifs along the promoter sequences. Specifically, we carry out the counting as follows.

1. Extract the promoter sequences of all annotated genes, say, − 1000 bp ~ + 200 bp from transcription start sites (TSS), In the case study of this article, we took the RefSeq mouse genome and annotation GRCm38.p4;
2. For each *cis*-motif, find its occurrences in one promoter sequence using the MATCH (version 2012) program provided by the TRANSFAC tool suite (version 2012) [1]. Repeat this step for all the motifs and all the genes.

The MATCH program has several options that respectively addresses sensitivity, specificity, etc. In this article, we select the option of minFN, which minimizes false negative rates.

### Variants

In cases when we are more concerned with the robustness and reliability of the transcriptional inferences, we might try some variant of the above modified BASE. For one example, we could substitute the sorted log-fold of gene expression vector {*d*_1_, *d*_1_,  … , *d*_*N*_}and/or TF binding affinity vector {*b*_1_, *b*_1_,  … , *b*_*N*_}by their ranks. Such a ranked-based BASE generally loses power of testing while gains robustness.

### Gene functional enrichment analysis with Wilcoxon rank test

Other than transcriptional inference, gene functional enrichment analysis (EA) is currently a direct and more popular method to interpret expression differentiation profiles into biology stories. That is, given a collection of gene subsets defined by KEGG pathways, or Gene Ontology (GO) including three related yet different aspects: biological processes, molecular functions and cellular components, we want to know which gene subsets have more occurances in the differentially expressed genes.

An intuitive EA approach is testing association between a gene subset and differentially expressed (DE) genes. To do this, we need to set a threshold for the definition of differentially expressed genes. When the expression difference of a gene is above the threshold, it is differentially expressed. Then we could arrange the counts of genes in a two by two contingency table according to their status of DE and their memberships of the gene subset. Consequently, the Fisher exact test could be applied to test the association. The rankings of the *p*-values from the association tests of all the gene subsets form an enrichment profile. However, such an EA procedure relies on the threshold for the definition of DE genes. And its selection is subtle and not straightforward.

In comparison, the most popular method, Gene Set Enrichment Analsyis (GSEA, [11]) does not requires predefined DE genes. It has been widely adopted in bioinformatic studies, even though its statistical properties such as power remains largely unknown.

Chao Cheng et al [12] proposed an idea to implement EA using the well-established Wilcoxon rank test of two samples, which has a good balance between statistical power and robustness.

The idea considers the gene ranks by their gene expression differences, and compares those falling inside a gene subset and those falling outside. The Wilcoxon rank sum test is applied to obtain a *p*-value under the no-difference hypothesis. Finally, we rank the *p*-values of all comparable gene subsets. Not only does this rank-based non-parametric method skip the definition of DE genes, but also gives robust conclusions. Its applications have led to several biological discoveries such as those in yeast aging [12], which were verified in [13, 14].

In this article, our aim is to make statistical inference of transcriptional regulation based on expression profiles and *cis-trans* binding strength*.* In addition to the statistical signfiances of the made inferences, it is very important to present biological justifications as well. On one hand, we could resorts to literature. On the other hand, we will use gene functional enrichment analysis with Wilcoxon rank sum test to confirm the inferences of regulations.

## Results

The central dogma essentially states that the *cis-trans* regulations are among primary causal factors of RNA transcript profiles. The modified BASE reversely infers the effective regulators from gene expression profile. In cooperation with other common bioinformatical analyses, BASE can help us obtain meaningful biological insights. Next we demonstrate how the modified BASE works by one expression data set, which was from the study of the the effects of EPA&DHA diets in mouse small intestinal epithelial cells.

Dietary polyunsaturated fatty acids (PUFA) were reported to be beneficial to human and animal health by modulating many important biological processes. However, the underlying molecular mechanisms were not completely clear yet. To find out key regulators involved in the effects induced by EPA&DHA diet, we applied our method to mouse expression data from the dietary intervention experiment conducted by Van Schothorstand and colleagues [15]. In this experiment, the mice were classified into two groups: the intervention group fed with an EPA&DHA diet containing 6% EPA and 51% DHA, and the control group fed with flax-seed oil (rich in alpha-Linolenic acid, ALA) as the only lipid source.

### Microarray data preprocessing

The gene mRNA expression values under the two diets were obtained by Affymetrix MOE430_2 GeneChip mouse arrays [15]. Each gene corresponds to one or several probe sets, and each probe set contains 11 probes. Instead of the Affymetrix default algorithm (MAS 5.0), which was used in [15], we took the Sub-array method to normalize the microarrays [16] to reduce the unwanted variations due to factors such as uneven hybridization and washing. Namely, we divide each array into subarrays of 50 by 50 probes. Within each subarrays, a piecewise linear relationship between the reference and target was estimated using least trimmed squares [17]. And this piecewise linear transformation was used to adjust the target sub-array. Adjacent subarrays overlap each other by 25 probes along both horizontal and vertical direction. Thus one probe may get multiple adjusted values, and we took their average as its normalized value. Finally we summarized the normalized probe values into probe set values by the PTR method [18].

**Fig. 2:**
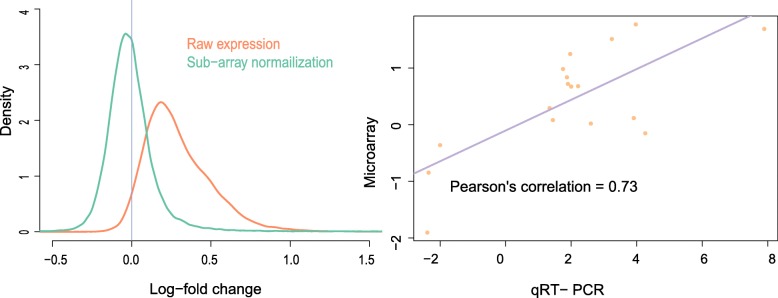
The validation of the expression profiles obtained by the microarray technique. Left: the kernel density of the gene expression differences between the EPA&DHA diet and control, with normalization and without normalization respectively. After normalization, both the bias, as measure by the closeness of the mode to zero, and the variation was reduced significantly. Right: the scatter plot of the expression fold changes of some significantly differentially expressed genes obtained by microarray and qRT-PCR, respectively. Their Pearson correlation coefficient is 0.73

The effectiveness of the normalization is illustrated on the left of Fig. 2 that showed the density plots of the probe set values before and after normalization. The correction of the bias is indicated by the reduced distance of the mode from zero. The reduction of variation is also obvious. As a matter of fact, the samples for microarray experiments are pools of multiple biological replicates. Thus the results represented a kind of average effect, and relative robust expression levels. Additional qRT-PCR experiments of some differentially expressed genes were reported in [15]. We displayed the scatter plot of the qRT-PCR results versus their microarray counterparts on the right of Fig. 2. The results were consistent, and their Pearson’s correlation coefficient is 0.73.

### Comparison with existing promoter analysis

The bioinformatics study in [15] included a multiple-step promoter analysis that detected seven transcription factors. The modified BASE differs from this method in several aspects. First, they used only 50 genes, which were obtained by several filters, for the promoter analysis while we used all genes (around 20,000 genes) included in the chip. Second, the promoter regions in their analysis were 650 bp upstream from TSS while our regions were from 1000 bp upsteam to 200 bp downstream. Thus we consider wider regulatory ranges. Third, they did not separate the TFs into up-regulation or down-regulation while we did. Fourth, they considered only those motifs whose factors were reported in at least 3 published articles (function word level B2), while we took a systematic approach and explored all available vertebrate motifs from TRANCFAC, in which the number of motifs exceeds 1400.

**Fig. 3:**
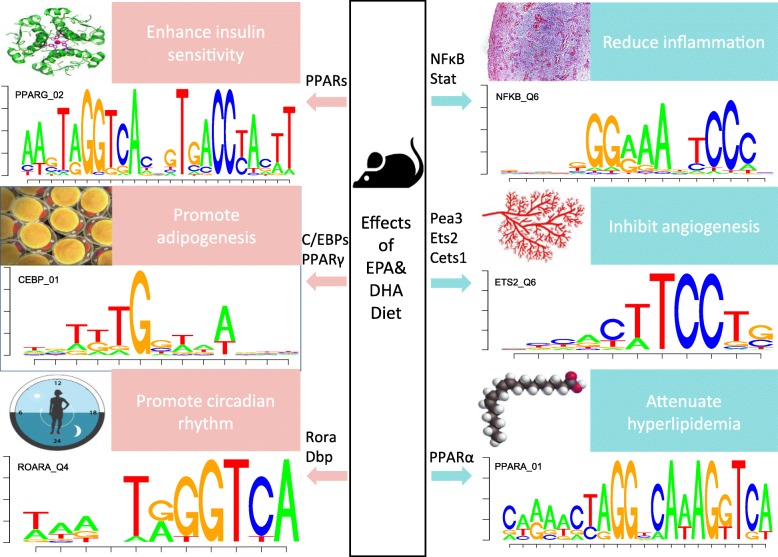
The effects of EPA&DHA diet in the mouse small intestine cells and corresponding TFs/motifs

We applied the procedure of modified BASE to the gene expression data and expected to find out the TFs driving the differentially expressed genes between the control and intervention groups. Compared with the naïve TF identification by promoter analysis conducted by van Schothorst et al in [15], our method detected all seven TFs in their analysis, including PPARα involved in fatty acid metabolism, NF-κB and Stat3 involved in inflammatory response, Dbp involved in circadian rhythm, dimerization partners Ahr and Arnt, and the zinc finger TF Sp1. Besides, we also detected many other novel meaningful regulators such as PPARγ involved in lipid metabolism, factors of the Ets family involved in angiogenesis and Rora, another types of regulators involved in circadian rhythm. The effects of EPA&DHA diet in mouse small intestine cells and corresponding TFs were summarised in Fig. 3. Some details of BASE inferences were shown in Table 1. Complete results of BASE were attached in Additional file 1. We highlighted some of the biological discoveries as follows.

**Table 1:** Partial inference results of the significant TFs/motifs in the exploration of EPA&DHA dietary effects

TFs	Motifs	P-values, upside^**^	P-values, downside^**^	Regulation^*^	Functions
PPARα	V$PPARA_01	0.00010	0.55	↑	Attenuate hyperlipidemia ^[24]^ Enhance insulin sensitivity ^[35, 36]^ Promote fatty acid metabolism ^[37]^ Regulate adipogenesis ^[19, 20]^
	V$PPARDR1_Q2	0.00030	0.37		
PPARγ	V$PPARG_01	< 0.00010	0.22	↑	
	V$PPARG_02	0.00010	0.17		
C/EBPs	V$CEBP_C	0.0049	0.69	↑	Induce adipogenesis ^[21–23]^
	V$CEBPA_Q6	0.0058	0.57		
	V$CEBPB_02	0.0039	0.66		
	V$CEBPD_Q6_01	0.0043	0.74		
	V$CEBPE_01	0.0006	0.97		
	V$CEBPG_Q6	0.0019	0.47		
NF-κB	V$NFKB_C	0.73	0.0039	↓	Reduce inflammation ^[38]^
	V$NFKB_Q6	0.33	0.0033		
Stats	V$STAT1_Q6	0.011	0.27	↑	
	V$STAT4_Q5	< 0.00010	0.26		
	V$STAT5A_02	0.0074	0.30		
Ets family	V$ETS2_Q6	0.91	0.011	↓	Inhibit angiogenesis ^[27, 28]^
	V$PEA3_01	0.33	0.0050		
	V$CETS1_01	0.40	0.0066		
	V$CETS2_02	0.46	0.036		
	V$ELF_02	0.17	0.0098		
	V$ELF4_02	0.17	0.0047		
	V$ELF5_03	0.76	0.011		
	V$ERF_01	0.43	0.0269		
	V$ERF_02	0.14	0.0084		
	V$ETV3_01	0.17	0.0083		
Rora	V$RORA_Q4	0.0035	0.34	↑	Enhance circadian rhythm ^[30, 31]^
	V$RORA2_01	0.00020	0.89		
Dbp	V$DBP_Q6_01	0.015	0.98	↑	
SP1	V$SP1_01	0.37	0.0099	↓	–
Ahr	V$AHR_01	0.058	0.0082	↓	–
Arnt	V$ARNT_01	0.23	0.013	↓	Reduce hypoxia ^[29]^

* ↑↓: The TF/motif was up/down-regulated in modified BASE.

** Tests with the up/down-regualted genes, 10,000 permutations.

### EPA&DHA promotes adipogenesis

Two well-known peroxisome proliferator-activated receptors, PPARα (*p*-value of its motif V$PPARA_01 = 1e-4) and PPARγ (p-value of its motif V$PPARG_01 < 1e-4), which both are ligand-activated TFs belonging to a superfamily of the nuclear hormone receptors, rank at top on the up-regulation side. Many downstream genes of PAPPγ are associated with adipogenesis. PPARs are able to sense fatty acid signals derived from dietary lipids and then are activated to mediate: (1) lipid transport in plasma; (2) lipoprotein uptake by living cells via the induction of apolipoproteins and lipoprotein lipase expression; (3) and intracellular fatty acid metabolism [19, 20]. Another important class of TFs in the adipocyte differentiation are C/EBPs (CCAAT-enhancer-binding proteins), including C/EBPA (*p*-value of its motif V$CEBPA_Q6 = 0.0058), C/EBPB (p-value of its motif V$CEBPB_02 = 0.0039) et al. In vitro and in vivo studies have demonstrated that each of them plays an important role in adipogenesis [21–23]. Thereby up-regulation of these TFs enhances adipogenesis.

### EPA&DHA attenuates hyperlipidemia

The activation of PPARα is directly induced by the intake of fatty acid (FA). It is known that PPARα, whose activation reduces hyperlipidemia, is highly expressed in intestinal epithelial cells. Rino Kimura et al’s research [24] revealed that DHA could increase FA oxidation and oxygen consumption rate, and decrease the secretion of triacylglyceride (TG) and apolipoprotein B (apoB), and hence could attenuate hyperlipidemia. As shown aboved, its binding motif V$PPARA_01 as well as V$PPARDR1_Q2 are significantly up-regulated.

### EPA&DHA inhibits angiogenesis

Angiogenesis is essential for normal development and homeostasis. However, unwanted angiogenesis has been implicated in a number of pathologic diseases, such as vaso-occlusive, psoriasis, arthritis, obesity and even tumor development [25, 26]. In the study, we discovered that dietary intake of EPA&DHA could result in decreased activities of several transcriptional mediators of angiogenesis. Assisted with the modified BASE, we found that several motifs were significantly down-regulated such as V$ERF_02 (*p*-value: 0.0084), V$ELF4_02 (*p*-value: 0.0047), V$ELF5_03 (*p*-value: 0.0108), V$ETV3_01 (p-value: 0.0083), V$PEA3_Q6 (p-value: 0.0052), and V$ETS2_Q6 (p-value: 0.0109). The ETS (E26 transformation-specific sequence) TF family, including Elf, Erf, Ets,Pea3 and some other subfamilies, have been implicated in vascular development and angiogenesis [27, 28]. The EPA&DHA diet results in down-regulation of the ETS family and inihiting the angiogenic factors.

### Hif − 1 signaling pathway

Angiogenesis is generally the downstream effect of the Hif-1 signaling pathway. Arnt is also identified as the beta subunit of the heterodimeric transcription factor, hypoxia-inducible factor 1. In the BASE result, the regulation of Arnt (*p*-value of its V$ARNT_01 = 0.0134) is significant on the down-side. Besides, the regulation of NF-κB and Stat3, which are involved in the Hif-1 signaling pathway, also played roles of down-side regulations. All these point to the down-regulation of Hif-1. In fact, it was suggested that polyunsaturated fatty acid induces a reduction in hypoxia in subcutaneous adipose tissue [29].

### EPA&DHA promotes circadian rhythm

Van Schothorst et al [15] reported a significant TF Dbp (D Site-Binding Protein) using their promoter analysis (2 out of the 50 genes they considered are targets of Dbp). The encoded protein of Dbp could bind DNA as a homo- or heterodimer and was involved in the regulation of some circadian rhythm genes [30]. In our BASE results, not only Dbp (*p*-value of its motif V$DBP_Q6_01 = 0.0153) but also RORA and RORA2 (RAR-related orphan receptor alpha, *p*-value of motif V$RORA_Q4 = 0.0035, p-value of motif V$RORA2_01 = 2e-4) were significantly up-regulated. RORAs also participated in the transcriptional regulation of some genes involved in circadian rhythm [31]. Put together the above two observations, we would rather infer that the EPA&DHA diet countributes to the maintaince of circadian rhythm.

### Other transcription factors

As a matter of fact, the BASE results include more transcriptional inferences other than the above factors, Many of them were not report in [15]. For example, Hnf4a is a known regulator induced by DHA and EPA [15], but it was not identified in [15]. Nevetheless, in the BASE results, Hnf4a’s regulation is significant on the up-side (*p*-value of its motif V$HNF4_Q6_01, V$HNF4A_02 < 1e-4). Hnf4a has been shown to interact with the biosynthesis of long chain PUFA [32], and the oxidative metabolites of PUFA in the fashion of specific ligand dependence [33]. This illustrates that the modified BASE inference is more systematic or comprehensive.

### Verification by gene functional enrichment analysis with Wilcoxon rank test

Gene functional enrichment anaylysis is a common practice in the bioinformatics research. We used the rank-based approach as descriped in Methods, to verify the TF regulatory activities we inferred. Several conclusions from the inference of TFs regulation with the modified BASE were verified by the enriched pathways or biological processes. A summary of the EA were shown in Table 2. In particular, we observe that several specific activities involved in lipid metabolism such as “PPAR signaling pathway”, “regulation of fatty acid oxidation” and “fatty acid metabolism” et al, were significantly up-regulated in the intervention group, which indicated that EPA&DHA increased lipid catabolism by up-regulating genes involved in long chain fatty acid beta-oxidation occurring in mitochondria and peroxisomes. Furthermore, the down-regulation of pathway “blood vessel development” verified our conclusion that EPA&DHA inhibited angiogenesis. And the up-regulation of several biological processes such as “intestinal immune network for IgA production” verified EPA&DHA induced inflammation. Detailed results Wilcoxon enrichment analysis were showed in Additional file 2.

**Table 2:** Partial conclusions from the inference of TFs regulation were verified by gene functional enrichment analysis

Conclusions	Pathways	P-values, upside^*^	P-values, downside^*^	Regulation
Enhance insulin sensitivity Promote adipogenesis Attenuate hyperlipidemia	PPAR signaling pathway (KEGG)	3.98e-8	≈1	↑
	Peroxisome (KEGG)	4.62e-11	≈1	
	peroxisome organization	2.99e-4	≈1	
Promote fatty acid metabolism	fatty acid metabolism (KEGG)	1.86e-9	≈1	↑
	cellular lipid metabolic process (GO)	3.49e-7	≈1	
	long-chain fatty acid metabolic process (GO)	9.54e-6	≈1	
	very long-chain fatty acid metabolic process (GO)	6.73e-5	≈1	
	regulation of fatty acid oxidation (GO)	5.30e-4	≈1	
	fatty acid beta-oxidation (GO)	1.17e-5	≈1	
Inhibit angiogenesis	blood vessel development (GO)	0.996	3.72e-3	↓
Reduce inflammation	intestinal immune network for IgA production (KEGG)	0.995	5.32e-3	↓

* Gene functional enrichemnt in the up/down-side with Wilcox rank sum test, see Method.

## Discussion

One pillar of systems biology is the systematic omic data from cell or tissues. It is a great challenge to develop statistical and computional methods that effectively integrate omic data and infer biological insights with significance. The scenario of expression profiles from samples of treatment and control plays the similar role as the two-sample problem does in statistical inference. In this situation, a typical analysis is the gene set enrichment analysis that helps us understand the biological activities between different experimental conditions. But how the TFs regulate these differentially expressed genes in response to environmental changes remain unclear. In this article, we introduced a computation-based, experimental condition-free measurement of TF binding affinity to boost the transcriptional inference method - BASE.

Of course, the complete inference of TF activities is challenged by many complications. We name a few as follows: (1) one gene may be cooperatively regulated by multiple upstream genes; (2) one transcriptional regulator may have positive or negative impacts on multiple downstream genes; (3) technical limitation of microarray and RNA-seq [34] techniques restrict us from getting accurate measurements of transcript quantities, particularly those of low abundance; (4) the existence of alternative splicing and other mechanisms increase the difficulty of TF activity inference. We did clone the BASE method to infer the regulations of microRNAs, see [6]. But definitely more efforts are needed to develop integrative frameworks of statistical inferences in the future.

In terms of TF binding affinities, so far we focus on the motifs in the nearby region of TSS, leaving out the distal enhancers regions, which play important regulatory roles as well. We did so because first the promoter regions are more straightforward than enhancers and second the TF bindings on promoter regions are more direct regulatory events for the transcription initiation. Of course, an elaborate computational model, which includes not only promoters but also enhancers et al, is worthy of being investigated.

In recent years, the new technology such as DNase-seq and ATAC-seq allow us to obtain the genome-wide openness status of DNA chromatins. It has been demonstrated that integration of chromatin accessibility data and motif occurrence data is much better than motif occurrence data themselves in terms of predicting gene expressions [4]. It is of great interests to develop accurate quantitative models of transcriptional regulations, taking into account of motif occurrence and other DNA information such as chromatin accessibility data in the future.

## Conclusions

In this article, we demonstrate that the cis-trans regulations underlying an expression differentiation profile can be effectively inferred statistically by the method BASE2.0 with an appropriate definition of TF-DNA binding strengths. Unlike the ChIP-seq data, we proposed a condition-free TF-DNA binding strength motivated by a probability model. It turns out that the binding strength of a cis-element by its interacting protein is approximately proportional to the corresponding motif frequency in the regulatory DNA regions. In an examplary study of DHA&EPA diet, we used a publicly available microarray data set to illustrate the effectiveness of the computational method. The inferred cis-trans regulations of of DHA&EPA diet are consistent with those reported in the literature, including PPARα and NFκB, respectively corresponding to enhanced adipogenesis and reduced inflammation. Moreover, we discovered enhanced RORA regulation of circadian rhythm, and reduced regulation by the ETS family.

## Additional files

Additional file 1: The completed BASE 2.0 results of each motif, defining the TF binding affinities as motif frequencies. (CSV 33 kb)

Additional file 2: Gene functional enrichment analysis with Wilcoxon rank test of KEGG and GO pathways. (XLSX 283 kb)

## Abbreviations

ALA: Alpha-linolenic acid
BASE: Binding association with sorted expressions
DE: Differential expression
DHA: Docosahexaenoic
EA: Enrichment analysis
EPA: Eicosapentaenoic
FA: Fatty acid
PUFA: Polyunsaturated fatty acid
TF: Transcription factor
TG: Triacylglyceride
TSS: Transcription start site
